# Supplementary material for: SILVR: Guided Diffusion for Molecule Generation
Source: J Chem Inf Model. 2023 Sep 19;63(19):5996–6005. doi: 10.1021/acs.jcim.3c00667 (PMC10565820; doi:10.1021/acs.jcim.3c00667)
Supplement: Supplementary file 1 — ci3c00667_si_001.pdf [file ci3c00667_si_001.pdf]

Supporting Information for SILVR: Guided Diffusion for Molecule Generation

Nicholas T. Runcie and Antonia S.J.S. Mey*

EaSTCHEM School of Chemistry, University of Edinburgh, EH9 3FJ, United Kingdom

E-mail: antonia.mey@ed.ac.uk

Summary of additional figures

The supporting information consists of a series of figures in addition to the figures in the main paper. Figure S1 contains the 2D structures of all fragments used in the experiments as references. In Figure S2 an example of two curated experiments is shown with different SILVR rates r_s. Next, Figure S3, shows on average how many molecules were not-fragmented in a set of 1000 samples for different SILVR rates. Figure S4 includes synthetic accessibility([1]) and QED [2, 3]. An example of how far atoms in the reference structure are displaced in the denoising-diffusion process is shown in an example in Figure S5 **A** for a whole molecule generated and in **B** for the heterocycle. A summary of experiments around the number of dummy atoms used in linker design experiments is seen in Figure S6 for $r_s = 0.005$ in A and $r_s = 0.01$ in B. The final Figure S7 shows a series of 2D samples from an uncurated list of samples, clearly showing examples of fragmented molecules.

Modified SILVR with dummy atoms

The SILVR protocol guides latent atoms according to a one-to-one mapping with reference atoms. This model is limited to reference molecules that somewhat overlap. Two fragments that are sufficiently far apart will not be successfully linked together. A universal EDM protocol for interpreting fragment data would ideally be able to do both fragment merging and linking.

Dummy atoms are latent space atoms that do not have a mapping to a reference atom, and instead are free to explore the latent space unguided. It was hoped that in the case of disconnected fragments, these dummy atoms would be able to form a linker. In practice, linker formation was observed both with and without dummy atoms. It was also observed that dummy atoms often populate unfilled valences as hydrogen atoms.

The total number of atoms used by the model was set as the sum of reference (n_r) and dummy atoms (n_d). The SILVR vector was created such that the first n_r indices held the value of r_S defined in the experiment protocol, and the subsequent n_d indices were set to zero so that SILVR would not be applied to the dummy atoms.

The centre of geometry for the reference coordinates was initially aligned at zero. Within the denoising loop, the EDM sampled new coordinates for all atoms. The new coordinates for dummy atoms were then added to the reference coordinates, and the modified reference coordinates were re-aligned at zero. The SILVR equation was applied as described in the main paper, only refining the atoms mapped to a reference. Through each iteration of denoising, the coordinates of the dummy atoms within the reference were continually updated, and the centre of geometry of these coordinates was re-aligned at zero. The total shift in the centre of geometry of the reference was tracked and subtracted from the final sampled molecule.

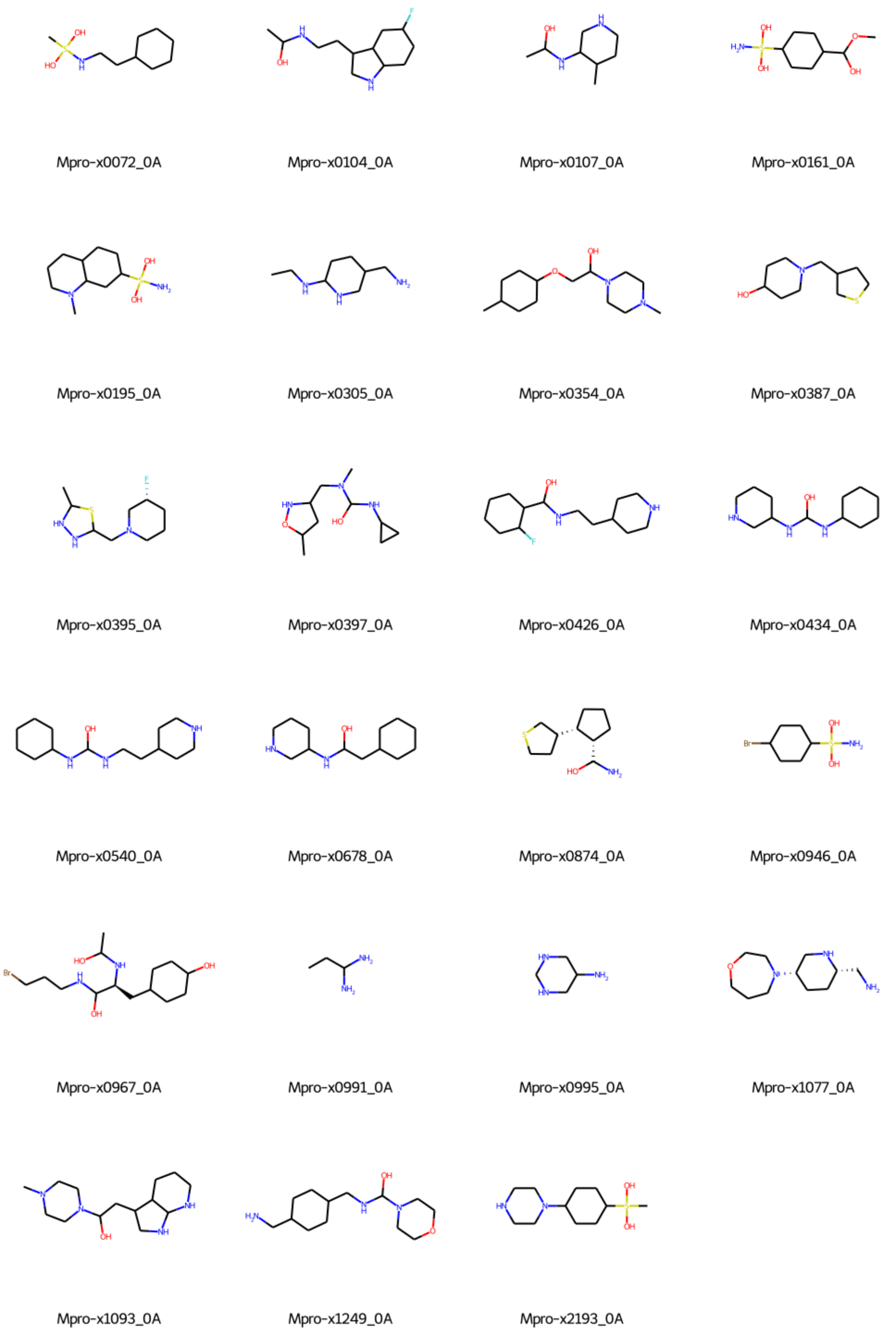

Figure S1: 2D Mpro structures from the moonshot dataset.

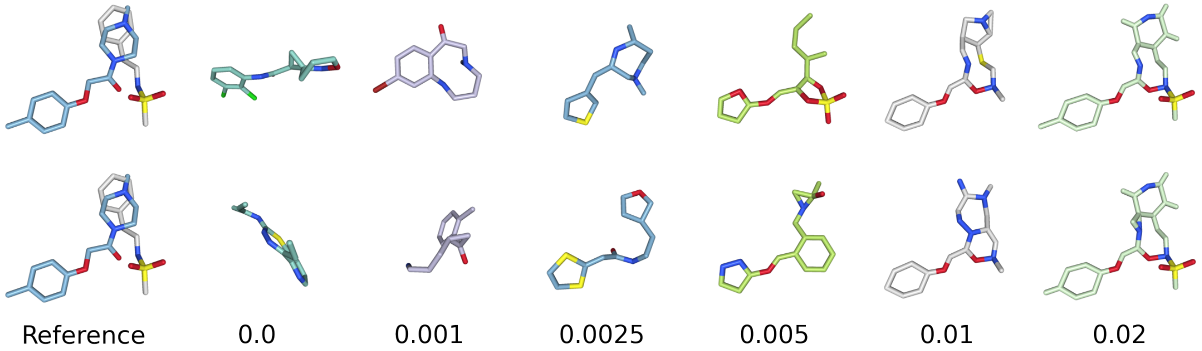

Figure S2: Two random samples from the same reference using different SILVR rates. All bonds were inferred from `XYZ` coordinates with OpenBabel. All bonds were visualised as single bonds and hydrogen atoms were deleted for clarity. Increasing SILVR rate results in sampled atom coordinates coming closer in space, and element type, to the reference while still resembling a truly molecular structure.

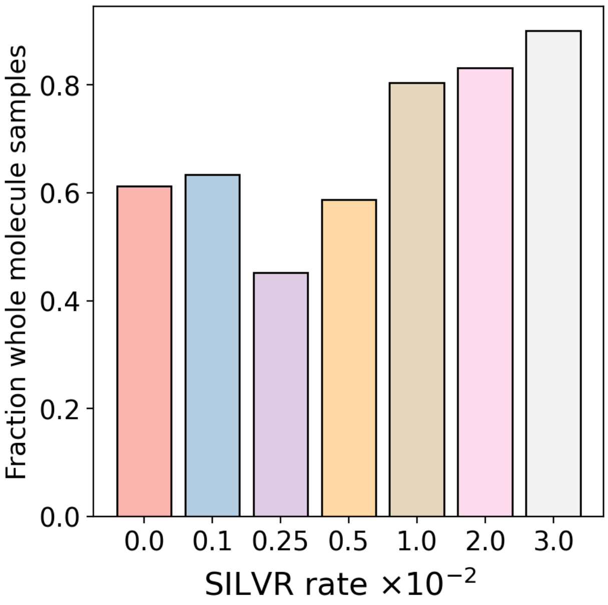

Figure S3: Fraction of molecules not fragmented with respect to the SILVR rate

References

(1) Ertl, P.; Schuffenhauer, A. Estimation of Synthetic Accessibility Score of Drug-like Molecules Based on Molecular Complexity and Fragment Contributions. *J. Cheminformatics* **2009**, *1*, 8.

(2) Bickerton, G. R.; Paolini, G. V.; Besnard, J.; Muresan, S.; Hopkins, A. L. Quantifying the Chemical Beauty of Drugs. *Nature Chem.* **2012**, *4*, 90–98.

(3) Wildman, S. A.; Crippen, G. M. Prediction of Physicochemical Parameters by Atomic Contributions. *J. Chem. Inf. Comput. Sci.* **1999**, *39*, 868–873.

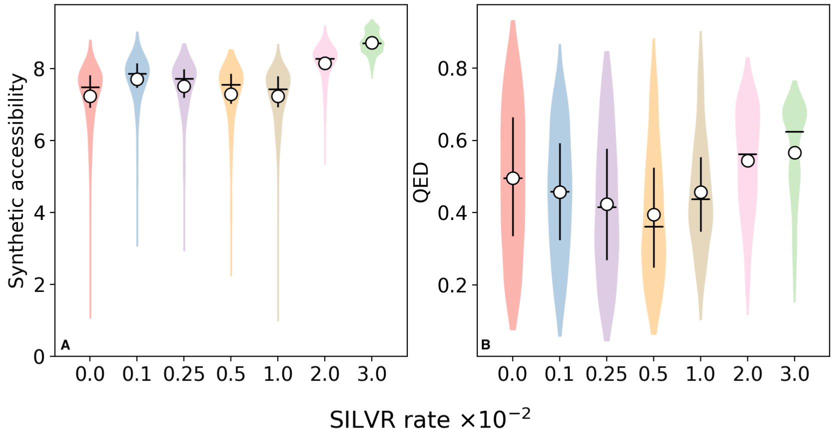

Figure S4: A: The synthetic accessibility score (SA score) estimates the synthetic feasibility of molecules based on fragments contributions. [1] This was calculated for all non-fragmented samples using an RDKit implementation of the scoring function SAScorer [1]. A lower score indicates an easier-to-synthesise molecule: most catalogue and bioactive molecules fall in the range 2-5, while a score greater than 7 represents the upper end of complexity for natural products. [1] B: The Quantitative Estimate of Druglikeness (QED) score combines a selection of descriptors such as molecular weight and calculated Log(P) to estimate the drug-likeness of a molecule. [2, 3] This was calculated using RDKit with default settings across all non-fragmented samples. A higher score indicates a more drug-like molecule.

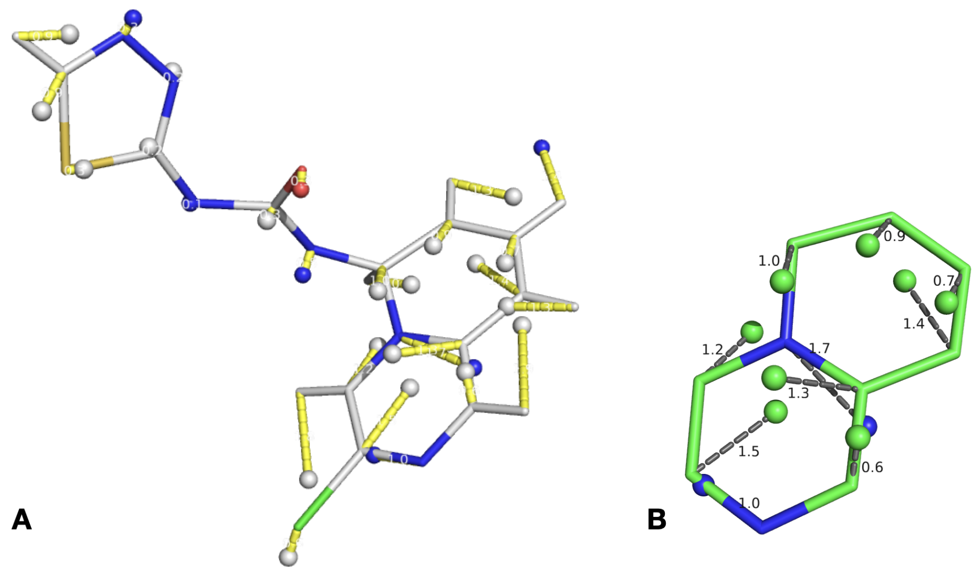

Figure S5: Example looking at the displacement of reference atoms after SILVR denosing. A: for the whole molecule. B: Zoom in, on the displacement of atoms in the 9aH-Pyrido[1,2-a]pyrazine heterocycle.

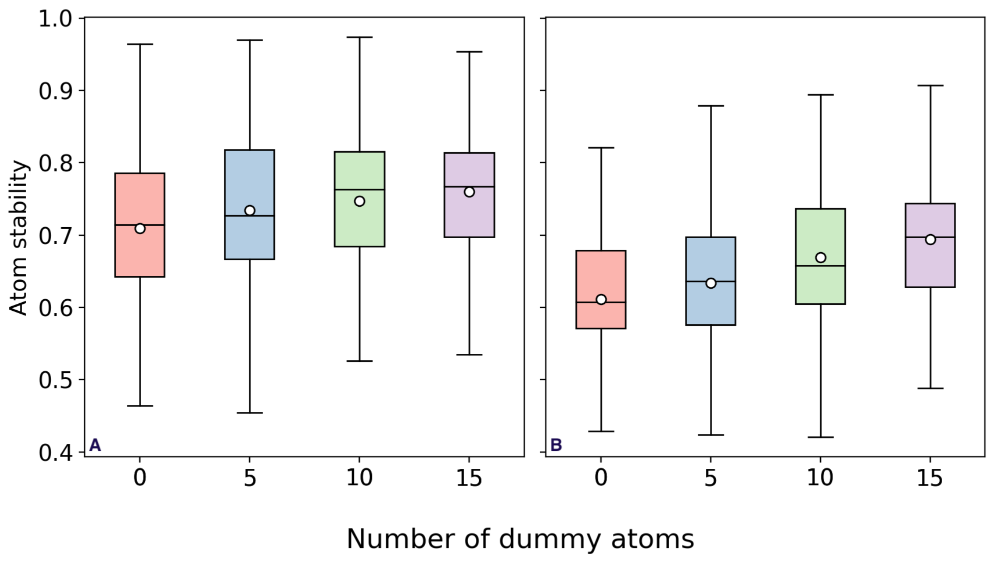

Figure S6: Effect of dummy atoms on the atom stability for linker design type experiment with reference fragments x0874 and x0397. A: $r_{\mathrm{S}} = 0.005$ and B:$r_{\mathrm{S}} = 0.01$. Lines represent sample median, circles sample mean.

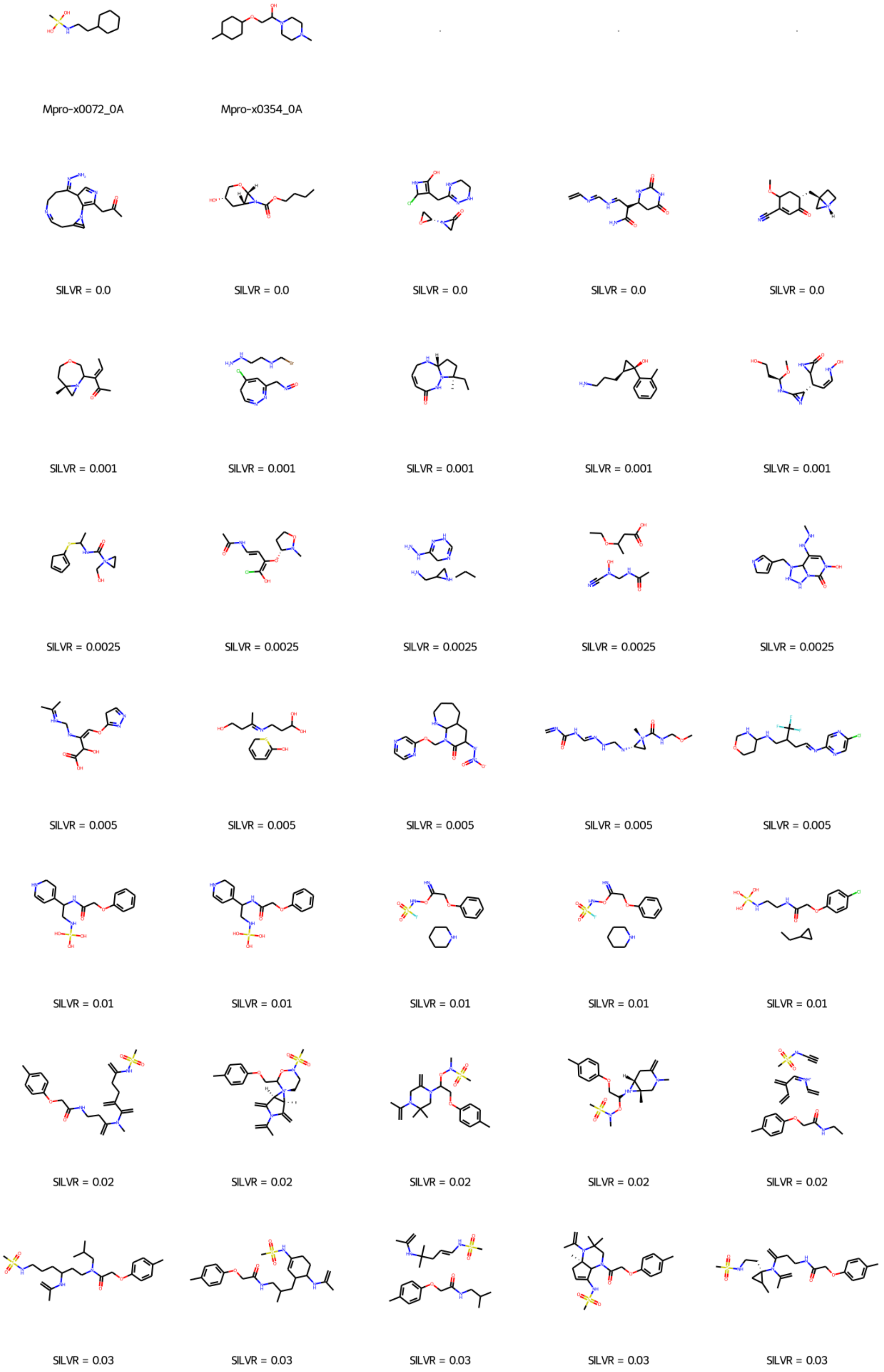

Figure S7: Uncurated samples, showing fragmentation of molecules based on input with two starting structures.
